# Automatic detection of pesticide residues on the surface of lettuce leaves using images of feature wavelengths spectrum

**DOI:** 10.3389/fpls.2022.929999

**Published:** 2023-01-26

**Authors:** Lei Sun, Xiwen Cui, Xiaofei Fan, Xuesong Suo, Baojiang Fan, Xuejing Zhang

**Affiliations:** College of Mechanical and Electrical Engineering, Hebei Agricultural University, Baoding, China

**Keywords:** pesticide residue, environmental protection, spectrum, rapid detection, vegetable leaves

## Abstract

The inappropriate application of pesticides to vegetable crops often results in environmental pollution, which seriously impacts the environment and human health. Given that current methods of pesticide residue detection are associated with issues such as low accuracy, high equipment cost, and complex flow, this study puts forward a new method for detecting pesticide residues on lettuce leaves. To establish this method, spectral analysis was used to determine the characteristic wavelength of pesticide residues (709 nm), machine vision equipment was improved, and a bandpass filter and light source of characteristic wavelength were installed to acquire leaf image information. Next, image preprocessing and feature information extraction were automatically implemented through programming. Several links were established for the training model so that the required feature information could be automatically extracted after the batch input of images. The pesticide residue detected using the chemical method was taken as the output and modeled, together with the input image information, using the convolutional neural network (CNN) algorithm. Furthermore, a prediction program was rewritten to generalize the input images during the prediction process and directly obtain the output pesticide residue. The experimental results revealed that when the detection device and method designed in this study were used to detect pesticide residues on lettuce leaves in a key state laboratory, the coefficient of determination of the equation reached 0.883, and the root mean square error (RMSE) was 0.134 mg/L, indicating high accuracy and that the proposed method integrated the advantages of spectrum detection and deep learning. According to comparison testing, the proposed method can meet Chinese national standards in terms of accuracy. Moreover, the improved machine vision equipment was less expensive, thus providing powerful support for the application and popularization of the proposed method.

## Introduction

Chemical approaches still play a dominant role in pest control in agriculture and forestry, and inappropriate application of chemical pesticides results in pesticide waste and environmental pollution (Pandiselvam et al., 2020; Anbarasan et al., 2022; Pandiselvam et al., 2022). Traditional artificial pesticide spraying is relatively extensive, while unmanned aerial vehicle-assisted pesticide spraying is greatly affected by environmental factors, such as wind (Zangina et al., 2021). The determination of pesticide residue quantity on the surface of crop leaves is a precondition for precise and targeted pesticide application (Tsagkaris et al., 2021). Excessive and non-uniform pesticide spraying can be reduced by accurately detecting crop leaf surface pesticide residues, thus protecting the environment and human health (Sun et al., 2021). Crop protection has evolved from the age of botanical pesticides and inorganic synthetic pesticides into the present-day era of organic pesticides, which are very effective at controlling pests. Chemical pesticides already account for 80% of pest control, but 3R phenomena (residue, resurgence, and resistance) have occurred due to the excessive reliance on them, thus influencing the whole environment (Dai et al., 2019). Starting with the precise detection of pesticide residues, research institutes have carried out technical studies regarding pesticide reduction and their precise application, exploring environmentally friendly and resource-saving crop planting methods that contribute to food security (Zheng and Xu, 2021).

Current pesticide residue detection methods mainly involve gas chromatography (GC) (Chen et al., 2021a), liquid chromatography (LC) (Zhou et al., 2021), and GC-mass spectrometry (GC-MS) (Li et al., 2020), which, although characterized by high detection sensitivity, strong specificity, and high accuracy, are associated with tedious sample preprocessing, high equipment costs, large instrument volumes, cumbersome data processing, and poor portability, thus failing to satisfy the desire for real-time fast pesticide residue detection (Nzarloo et al., 2021). In recent years, various spectroscopic methods have brought about substantial advancement in the field of pesticide residue detection (Lin et al., 2020). Teixeira combined enhanced Raman scattering spectroscopy with paper-based gold nanoparticles to detect pesticide residues on mango skin (Teixeira et al., 2020), and Guo L Q et al. successfully detected fluorescent 4-methyl umbelliferone using fluorescence spectrometry (Guo et al., 2017). However, these instruments are mainly used for detecting pesticide residues in an aqueous solution system, and there are few portable instruments that can directly detect pesticide residues on the surface of plant leaves; therefore, the rapid detection required by the agricultural industry cannot be achieved using these methods. When the hyperspectral technique is used to detect pesticide residues, differences in the internal physicochemical properties of the target can be determined to some extent. The spectral curves of crops sprayed with different pesticides are varied, and besides, regression models of spectra, pesticide type, and concentration can be established by acquiring the scalar values of parameters based on traditional chemical detection methods, facilitating pesticide residue detection and analysis. Gui J S et al. qualitatively detected pesticide residues on the surface of broccoli using a hyperspectral imaging technique that involved preprocessing, the selection of characteristic wavebands, and the construction of a prediction model (Gui et al., 2018).

Sun J and Cong S L established a support vector machine (SVM) and an optimization model to qualitatively analyze pesticide residue types on lettuce leaf surfaces using the hyperspectral imaging technique, and then quantitatively detected pesticide residues using PLS-SVM (Sun et al., 2018). The detection of pesticide residues on the leaf surface using the spectral method can be easily affected by internal and external factors, such as light irradiation angle (Meng et al., 2014; Gong et al., 2016), leaf surface smoothness (Zheng et al., 2016), and leaf position (Yang and Wu, 2009), which give rise to inaccurate results. Therefore, it is difficult to provide precise pesticide residue detection, and, as such, the development of a portable detecting instrument has been scarcely explored (Cai et al., 2007). For the detection of pesticide residues in fruits and vegetables, establishing a relationship model between the detection object and the pesticide residue will be of great significance in terms of detection accuracy (Li and Peleato, 2021; Lu et al., 2021; Nazarloo et al., 2021). Traditional modeling methods, such as multiple linear regression (MLR) and the BP neural network, are increasingly unable to meet researchers’ demands for prediction accuracy. In recent years, the deep learning method, based on big data size, has provided a powerful guarantee of precise modeling prediction (Zhu et al., 2020; Chen et al., 2021b; Nie et al., 2021). The convolutional neural network (CNN)-based deep learning algorithm has been extensively considered and applied by experts and scholars within the industry (Yan et al., 2021).

The novelty of this study is as follows:

1. The advantages of spectral analysis and machine vision detection of pesticide residue on crops, and low cost, were combined in this study.
2. By using the CNN algorithm as the training method, this study integrated the advantages of spectrum detection and deep learning.
3. Given the excessive modeling workload associated with traditional complex image processing methods, an automated training and detection program was developed; therefore, training and detection could be automatically extracted after the batch input of images, allowing efficient and rapid pesticide residue detection.

For the purposes of detecting pesticide residue on the surface of lettuce leaves in this study, the optimal characteristic wavelength of imidacloprid pesticide residue was acquired using IR spectroscopy. Next, improved machine vision equipment was used to capture images under the characteristic wavelength and extract their feature information. Afterward, the detected pesticide residue was modeled using the deep learning algorithm, i.e., CNN, to obtain the relationship model between the feature information of photos and pesticide residue under the characteristic waveband. Next, the CNN automatic image modeling method was put forward and the data preprocessing and extraction programs were merged with the packaged model. The model was then able to be rapidly established by importing the images. The result could be directly obtained by inputting one image into the model during each prediction process. Therefore, the modeling process was greatly simplified, modeling accuracy was improved by increasing the sample size, and the goal of rapid and accurate pesticide residue prediction in crop leaves was achieved, fulfilling the ambition of protecting the environment and human health, lowering equipment costs, and making the large-scale promotion of a tractable detection device and method possible.

## Experimental design

For detecting pesticide residues on the surface of lettuce leaves, the optimal characteristic wavelength of imidacloprid pesticide residue was obtained using IR spectroscopy. The machine vision equipment was then improved, and a bandpass filter and the light source of characteristic wavelength were installed to acquire leaf image information under the light of characteristic wavelength. The details of this process are described in this section.

### Experimental materials

**Figure 1:**
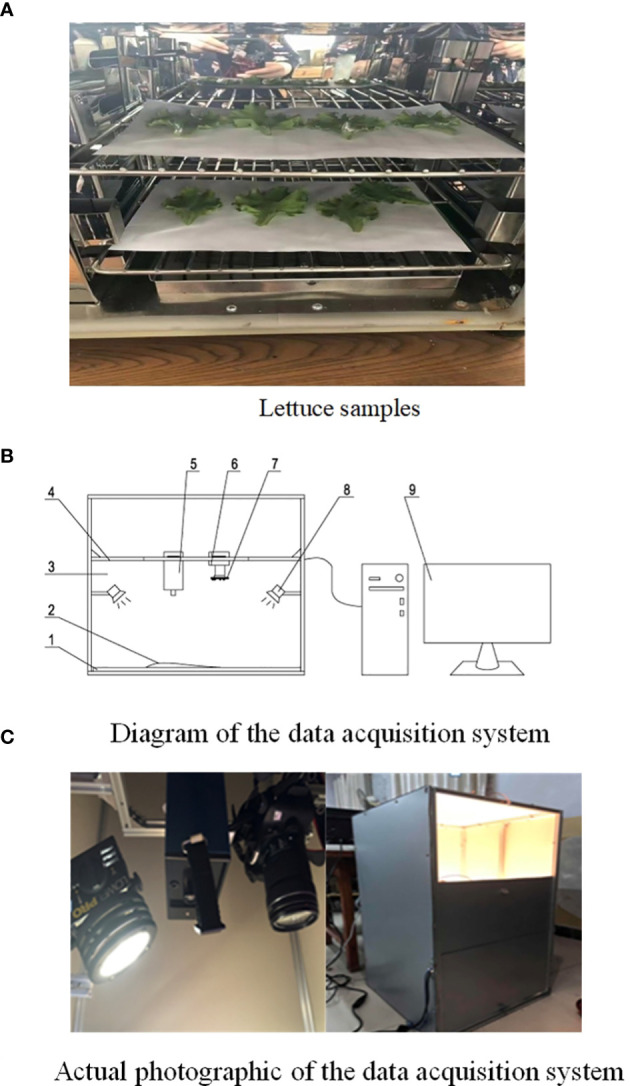
Sample data collection. **(A)** Lettuce samples. **(B)** Diagram of the data acquisition system. **(C)** Actual photographic of the data acquisition system.

Imidacloprid is a common pesticide that is harmful to human health, and was banned in China in 2020. However, many farmers still use imidacloprid, so we used it in our experiments. Imidacloprid with 70% active ingredient was diluted into a 10% solution using clear water; four dilutions (1:500, 1:800, 1:1500, and clear water [used as a control]) were prepared for sample treatment. A total of 120 lettuce leaf samples of similar size and free of surface damage were chosen, and divided into four groups (30 leaves per group), according to the concentration of pesticide to be sprayed in the validation experiment. Before the experiment, the sample surfaces were wiped using clean semiwet towels and sequentially labeled. The front and back surfaces of samples in different groups were sprayed with pesticides at the same dosage. Subsequently, all samples were placed in a shady, cool, and ventilated environment for 48 h. After new and old leaves were eliminated, leaves at the same position on each lettuce plant that were minimally different in leaf area, with no disease spots on the leaf surface, were equally divided into 10 portions to make the samples similar, and then preserved in sealed and labeled plastic bags. Some samples are shown in **Figure 1A**.

### Spectral information acquisition device

A diagram of the data acquisition system is shown in **Figure 1B** and features the following components:

1, objective table; 2, sample; 3, darkroom; 4, bracket; 5, spectrometer; 6, camera; 7, light source under feature wavelengths; 8, halogen lamp; 9, computer.

As shown in **Figure 1C**, the dark room was set up as the hardware platform of the detection system and to obstruct the penetration of external natural light. The spectrometer or image acquisition device were used to acquire leaf sample information only under the light source of the detection system to avoid disturbance from the external environment. The spectrometer and target object were placed so that the detection object was 20 cm from the spectrometer, and the object stage was fixed so that the sample could be fully covered by the viewing field. During the experimental process, the spectrometer was kept perpendicular to the sample, the field of view was set, and the halogen lamp (35 W, 230 V) was adjusted so that it was at least 25 cm from the spectrometer to prevent heat damage to the front-end camera of the spectrometer. The parameters of the acquisition device are listed in **Table 1**.

**Table 1:** Main equipment list for the hardware platform of the detection system.

Equipment	Specification and type
Whiteboard for calibration	15 mm×15 mm×600 mm
Spectrometer	320-1100 nm
Composite-light-source	235 W-230 V
Light source under feature wavelengths	Customized light source
Camera	Canon EOS70D

## Data acquisition and processing

Using the spectrometer, we acquired the spectral reflectivity of individual leaf samples. Data acquisition and modeling were implemented through the following steps:

1. Use the spectrometer to acquire the spectral reflectivity of each leaf sample under different pesticide residues.
2. Preprocess spectral reflectivity information (the processing is described subsequently).
3. Calculate the spectral characteristic wavelength of imidacloprid pesticide residue in lettuce leaves.
4. Take photos of samples in different groups using the camera installed with a light filter and light source of characteristic wavelength (the device was refitted in this study, as described subsequently).
5. Detect pesticide residues in the photographed samples.
6. Extract the average gray value, three-component mean, three-component standard deviation, and three-component coefficient of variation of the sample picture as the model input, and the pesticide residue as the output to establish and train the mathematical relationship model.

### Spectral data processing

The spectral reflectivity acquisition device used in this study was a PSR-1100F (Spectral Evolution, USA) spectrometer, with a wavelength range of 320-1100 nm, 5 nm precision, and 1 nm resolution. DAR Winp software was used to collect spectral data and change their format from SED to CSV. Then MATLAB 2018 was used for data processing. The original spectral data were preprocessed by combining standard normalization and SG convolutional smoothing to eliminate the effects of dimension, variable size, and value, and remove distortion data.

As different pesticide residues could not belong to the same order of magnitude, the input data had to be normalized before the mathematical model was established. The normalized mapping formula was as follows in Equation (1):


(1) $$f:x\to y=\frac{x-x_{\min}}{x_{\max}-x_{\min}}$$

*x*, *y*∈*R^n^*;*x_min_* =*min*(*x*);*x_max_* =*max*(*x*). *x* is the original data, *y* is the normalized data.

Smoothing filtering was implemented in this study *via* SG convolutional smoothing, which improved the smoothness of the hyperspectral surface and reduced noise. This method was an improvement on mobile smoothing. Application needs in multiple scenarios could be satisfied by selecting different window widths. The calculation process was as follows: the filtering width was set as *n*=2*m*+1, and the polynomial fitting of measurement points *x*=(-*m*,-*m*+1,0,…0,1,…*m*-1,*m*)was implemented within the window using a polynomial with *k*-1 orders, as seen in Equation (2):


(2) $$y=a_{0}+a_{1}x+a_{2}x^{2}+\cdots +a_{k-1}x^{k-1}$$

*a*, polynomial coefficient.

Hence, there are *n* such equations to form a set of *k*-element homogeneous equations. To ensure a solution to this equation set, *n* should be greater than *k*, and the fitting parameter *A* is determined by least squares fitting, as seen in Equation (3):


(3) $$\left(\begin{array}{c} y_{-m}\\ y_{-m-1}\\ \vdots \\ y_{m} \end{array}\right)=\left(\begin{array}{cccc} 1 & \text{-m} & \cdots & {\left(-m\right)}^{k-1}\\ 1 & -m+1 & \cdots & {\left(-m+1\right)}^{k-1}\\ \vdots & \vdots & \vdots & \vdots \\ 1 & m & \cdots & m^{k-1} \end{array}\right)\left(\begin{array}{c} a_{0}\\ a_{1}\\ \vdots \\ a_{k-1} \end{array}\right)+\left(\begin{array}{c} e_{-m}\\ e_{-m+1}\\ \vdots \\ e_{m} \end{array}\right)$$

*m*, *e*, coefficient to be determined by the least squares method.

The above equation is expressed in the following matrix form:


(4) $$Y_{(2\text{m}+1)\times 1}=X_{(2m-1)\times k}\cdot A_{K\times 1}+E_{K\times 1}+E_{(2m+2)\times 1}$$

*Y*,*X*,*A*,*E*, matrix form of *y*,*x*,*a*,*e*.

Where the solution obtained through the least squares method of *A* is:


(5) $$A={\left(X^{T}\cdot X\right)}^{-1}\cdot X^{T}\cdot Y$$

The model prediction value or filtering value of *Y* is:


(6) $$Y=X\cdot A=X\cdot {\left(X^{T}\cdot X\right)}^{-1}\cdot X^{T}\cdot Y=B\cdot Y$$

(7) $$B=X\cdot {\left(X^{T}\cdot X\right)}^{-1}\cdot X^{T}$$

**Figure 2:**
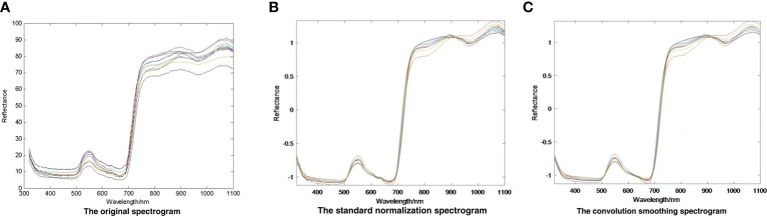
Data processing of spectral reflectance. **(A)** The original spectrogram. **(B)** The standard normalization spectrogram. **(C)** The convolution smoothing spectrogram.

After the spectral information was acquired, the original data, standard normalized data and the data after SG convolutional smoothing were depicted (**Figure 2**).

As shown in **Table 2**, to find the best preprocessing scheme, we used eight different methods to build a partial least squares (PLS) model for spectral data and compared it with the effect of model prediction. The eight kinds of spectral data include original spectral data, spectral data preprocessed by standard normalization, multivariate scattering correction, first derivative, convolution smoothing, convolution smoothing and first derivative, convolution smoothing and second derivative, and mean centralization. Most of the correlation coefficients of PLS modeling sets based on the preprocessing of the original spectrum were higher than the correlation coefficient of original spectral band modeling sets (0.6327), whereas the correlation coefficient of derivative modeling sets was 0.6218, lower than that of the original spectral band modeling, indicating that the derivative method was not applicable. According to the modeling effect, data modeling after convolution smoothing pretreatment can better reflect pesticide residues in lettuce leaves. Therefore, using spectral data preprocessed by convolution smoothing, the predictive effect of PLS modeling was better than the data obtained by the method. With the preprocessing method of convolution smoothing, Rc and Rp were up to 0.81332 and 0.7936, respectively, and SEC and SEP were up to 0.0743 and 0.0578, respectively. Compared with the original data, modeling accuracy was higher after using this method. Based on the above analysis, this study used the preprocessing method of convolution smoothing to extract feature wavelengths.

**Table 2:** Partial least squares modeling comparison of original spectral data and preprocessed data.

Preprocessing method	Modeling set		Prediction set	
	Rc	SEC	Rp	SEP
Original spectrum	0.6327	0.0664	0.6347	0.0436
Standard normalization	0.7321	0.0632	0.7562	0.0432
Multivariate scattering	0.6903	0.0639	0.6703	0.0562
First derivative method	0.6218	0.0692	0.6156	0.0593
Convolution first order differential	0.6593	0.0693	0.6892	0.0673
Convolution second order differential	0.7843	0.0621	0.7729	0.0697
Convolution smoothing	0.8132	0.0743	0.7936	0.0578
Mean value centralization	0.7689	0.0539	0.7539	0.0562

**Figure 3:**
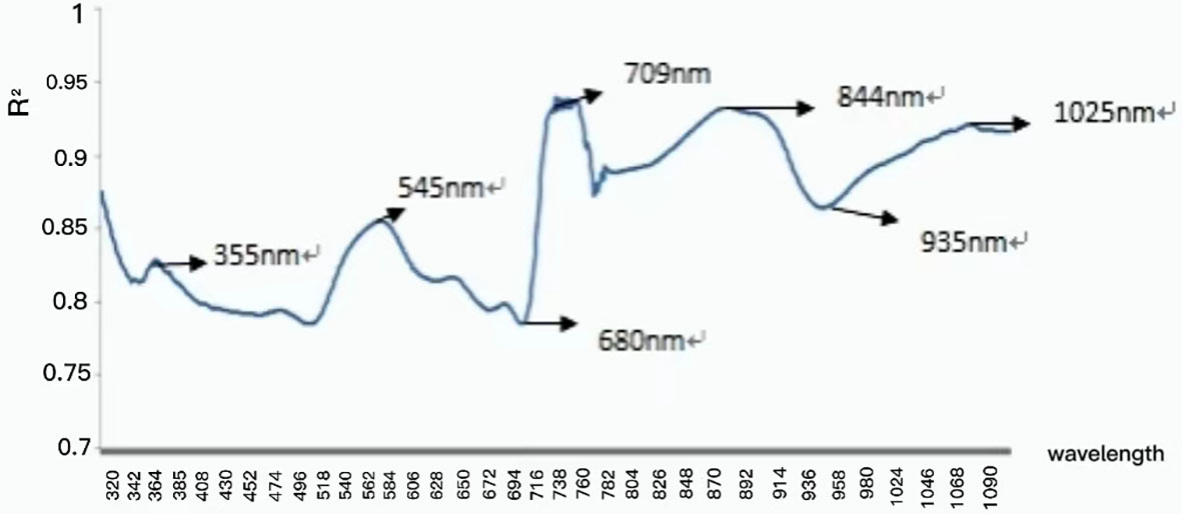
Correlation between logarithmic spectral reflectance and moisture content of drying base.

**Figure 4:**
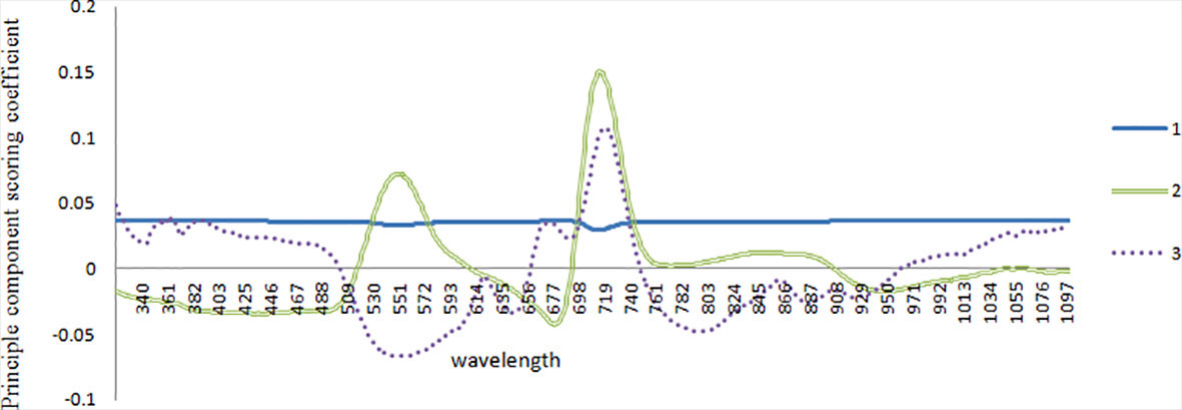
Correlation between the standard quantity coefficient and the wavelength coefficient with PCA.

First, the characteristic wavelength was extracted using the correlation coefficient method. Further data processing was also performed using this method, after data obtained by spectral preprocessing was logarithmically (lg1/λ) transformed. As shown in **Figure 3**, data processing revealed that the correlation coefficient R^2^ reached as high as 0.9 within the waveband of 725 nm, and of particular note, reached the maximum value of 0.94 at 709 nm. Second, the characteristic waveband with the highest correlation coefficient, highest scoring coefficient, and strongest comprehensive capacity was taken as the characteristic central light source by combining the principal component analysis (PCA). By standardizing the original light reflectivity data under different pesticide residues, the results showed that the proportion of variance sum of the first three principal components y_1_, y_2_, and y_3_ in the total variance reached as high as 95.72%, the total variance of the variance sum of the three principal components accounted for 97.72%, and only a small quantity of information was missing, namely, the information of original indexes was basically reserved. Each principal component was a linear combination of all the original variables, the absolute value of scoring coefficient decided the comprehensive capacity of each index in the principal component, and in this linear combination, the principal components were mutually uncorrelated. The data processing effect is shown in **Figure 4**. The first principal component nearly averagely integrated all the original variables, with a high scoring coefficient, whereas the second and third principal components were weaker than the first, manifesting that the variables within this waveband were of relatively strong comprehensive capacity.

The characteristic wavebands 355, 545, 680, 709, 844, 935, and 1025 nm were screened out using the correlation coefficient method and PCA, according to the correlation coefficient diagram and reciprocal diagram of the original standard coefficient and wavelength coefficient with PCA. Least squares modeling was implemented according to the extracted characteristic wavelength, and the characteristic waveband after convolutional smoothing preprocessing modeling effect and full-waveband modeling effect were compared (**Table 3**).

**Table 3:** Modeling results of full-wave band and characteristic waveband with optimal pretreatment.

	Rc	SEC	Rp	SEP
Full-waveband modeling a	0.8132	0.0743	0.7936	0.0578
Characteristic waveband modeling b	0.8234	0.06732	0.8052	0.0742

Full-waveband modeling a and characteristic waveband modeling b were implemented under full waveband. The fitted correlation coefficients of the modeling sets were 0.8132 and 0.8234, respectively, and 0.7936 and 0.8052, respectively, for the prediction sets, with standard deviations of 0.0578 and 0.0742, respectively. The comparison showed that the characteristic waveband modeling effect was higher than the full-waveband modeling effect, and characteristic waveband modeling prediction accuracy was higher.

Comprehensive analysis of the correlation coefficient method and PCA suggested that spectral reflectivity at 709 nm had the highest correlation with the pesticide residue, and had the highest scoring coefficient, indicating that the principal components showed the strongest comprehensive capacity at 709 nm. Hence, 709 nm was selected as the characteristic wavelength, the corresponding characteristic light source was chosen according to the characteristic waveband, and image information under the characteristic light source was extracted to detect pesticide residues on leaves.

## Detection device and method

As mentioned previously, each leaf sample was divided into 10 portions according to the field of view of the image acquisition equipment, so the sample size was enlarged to 1,200 for the large sample size data analysis. All parts of each leaf sample were considered in the established model.

### Acquisition device

**Figure 5:**
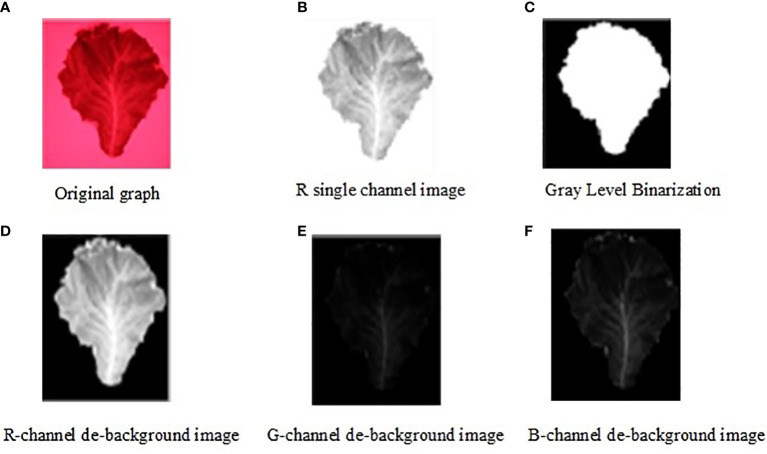
Targeted image extraction process during the characteristic wavelength spectrum. **(A)** Origin graph. **(B)** R single channel image. **(C)** Gray Level Binarization. **(D)** R-channel de-background image. **(E)** G-channel de-background image. **(F)** B-channel de-background image.

A Canon EOS70D camera (shutter speed, 30-1/8000; ISO, 100-25600; Canon Japan) was used for image acquisition. Sample images were obtained with an ISO setting of 12800 and shutter speed of 1/6000s. After the built-in light filter was removed from the camera lens, the customized 709 nm bandpass filter was installed in the lens to filter light from other wavebands. Additionally, eight 709-nm characteristic light sources were customized and uniformly distributed around the front-end lens of this camera. The lettuce leaves were shot using this tailor-made acquisition device; the characteristic light sources enhanced the irradiation on the leaf surface at a light wave of 709 nm, the bandpass filter filtered lights at other wavebands in natural light, and only the leaf images under the characteristic wavelength of 709 nm were acquired, thus enhancing modeling and detection accuracy. The images taken during the characteristic wavelength spectrum are shown in **Figure 5**.

### Modeling and model training

In this study, the leaf images acquired by the acquisition device were taken as the input. Four characteristic parameters—average gray value, three-component mean, three-component standard deviation, and three-component coefficient of variation—of the leaf images were extracted as the model input, and the pesticide residues on leaves detected by the State Key Laboratory of North China Crop Improvement and Regulation were taken as the model output. Ultra-high performance LC-tandem MS was performed, in line with national standards, in the State Key Laboratory with third party inspection. The general modeling steps were as follows:

1. Import the images acquired by the camera (using the software provided by the Canon).
2. Image preprocessing (target area extraction, image expansion corrosion, etc.).
3. Import images into the program to obtain intermediate data: average gray value, three-component mean, three-component standard deviation, and three-component coefficient of variation as the model input (these parameters are treated as the direct input).
4. Train the model input and laboratory-determined pesticide residues in Equation (3) to obtain the model (the model will be packaged in the program).

After the data in Equation (1) were obtained, the program was compiled to merge Equations (2)-(4) into the main program to facilitate the automatic implementation of three steps: batch preprocessing, extraction of model input, and pesticide residue modeling after the images were imported. Furthermore, the manual workload was reduced so that modeling accuracy could be improved by increasing the training samples.

Leaf images were three-channel RGB color images with a size of 6×6×3 channels, and the size of the convolution kernel was 3×3, i.e., a convolution kernel with three color channels, and thus a 4×4 characteristic pattern was generated. Next, gray processing of the RGB image was carried out to obtain the average gray value of the leaves, and the convolution kernel was converted into a single-channel convolution kernel to obtain the average gray value, three-component mean, three-component standard deviation, and three-component coefficient of variation. Appendix A shows the automatic data processing component in the main program, including steps (1)-(3).

The model training part of the main program only needed to invoke the results of the automatic processing component, thus realizing the one-key automatic operation of steps (1)-(4). First, the four groups of numerical values obtained in Equation (3) were standardized, the weight matrix was initialized, and the normally distributed noise with a standard deviation of 0.1 was added to improve training accuracy. The bias was initialized, some small positive values were added to avoid death nodes, and their tf.constant function was returned to the shaped matrix, with a function value of 0.1. When the pooling layer was defined, padding was selected for one stride each time, i.e., strides[1]=strides[2], to obtain more image information. To reduce the parameters and further mitigate system complexity, sparsification processing was carried out for the parameters *via* pooling; maximum pooling was adopted, and the size and step of the pooling kernel function were 2×2 and 2, respectively. The original data were converted into 6×6 two-dimensional images using x_image.

As the original RGB image underwent gray processing, the number of channels was set as 1. The first convolution layer was added, the size of convolution kernel was 2×2, the number of image channels was 1, the number of convolution kernels was 16, and the corresponding bias was 16. Next, the second convolution layer was added, the size of the convolution kernel was unchanged (2×2), the number of channels was 16, the number of convolution kernels was 32 (the number of convolution kernels was increased by multiples of 16), and the bias was set as 32. The third convolution layer, which was a fully connected layer, was added, and 4×4 three-dimensional images with a height of 64 were stretched into a one-dimensional array 512 in length. The output layer was added, the one-dimensional array was compressed into an array with a length of 1, and the bias was set as 1.

The rectified linear unit (ReLU) layer executed nonlinear mapping of the convolution layers, and the calculation formula was as follows (Equation 8):


(8) f(x)=max(0 x)

**Figure 6:**
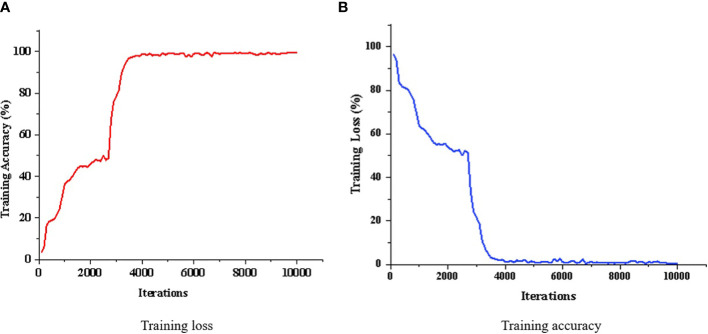
Training results. **(A)** Training loss. **(B)** Training accuracy.

The proportion of training sets to test sets was set as 8:2, the learning rate as 0.01, and the number of training times as 10,000, and one loss value was exported every 100 times of training. The sample training results are displayed in **Figure 6**. As the number of training times was increased, the loss value was continuously reduced, approaching 0, while the accuracy continued to rise until reaching close to 100%, thus proving the favorable modeling effect of the proposed method. The model was saved after being established, and was directly fetched for the prediction element. The lettuce leaf images were then imported, and imidacloprid pesticide residues on the leaf surface could be directly obtained.

**Figure 7:**
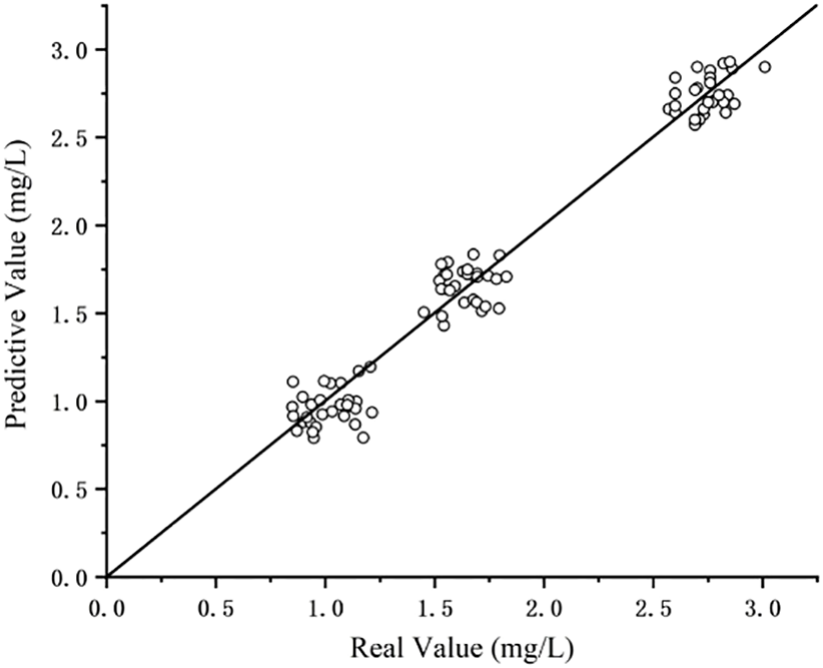
Test set fitting diagram.

The precision of the modeling test data was verified. The curve fitting can be seen in **Figure 7**. The coefficient of determination (R^2^) of equation was obtained as 0.969 and the RMSE value was 0.037, verifying the high accuracy and favorable convergence of the proposed modeling method. The detection results are as shown in **Table 4**. During testing, the press was 0.331, RMSE was 0.086mg/kg, and the MAE was 0.051mg/kg. According to Chinese testing standards (State Bureau of Technical Supervision, 2021), the maximum residue limit for pesticides in lettuce is 3mg/kg, and the detection accuracy is 0.15mg/kg. Therefore, the method proposed in this paper meets Chinese standard accuracy requirements.

**Table 4:** Detection results of the method proposed in this study.

Parameter	Training	Testing
Press	0.407	0.331
RMSE(mg/kg)	0.037	0.086
MAE(mg/kg)	0.022	0.051
R^2^	0.969	0.901

To verify the effectiveness of the proposed method at detecting pesticide residues, a comparison validation was made between the predictive values and real values. Validation procedures were as follows:

Validation preparation: 30 lettuce leaves were selected and sprayed with imidacloprid pesticides of different concentrations. The samples were then naturally dried. Predictive values and real values will be compared under the same concentrations.

**Figure 8:**
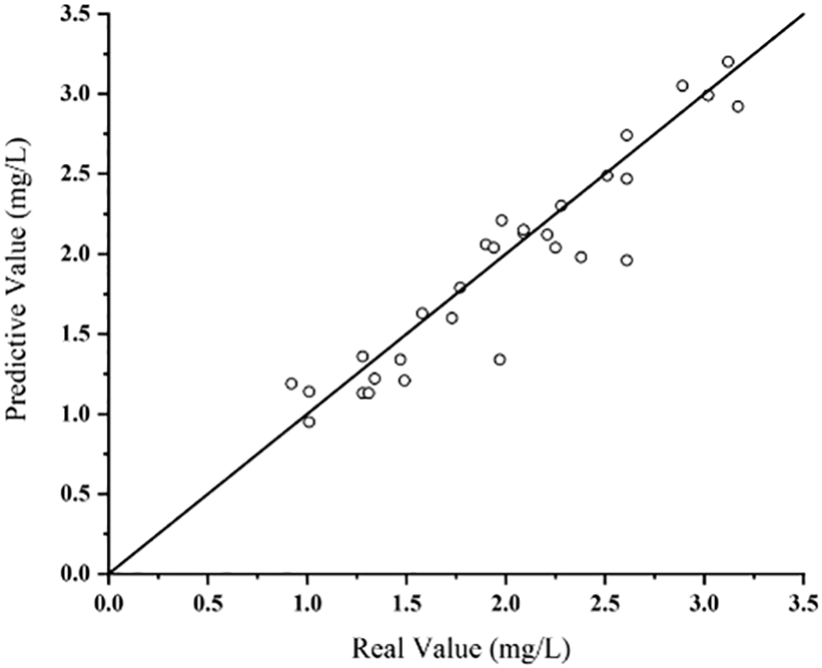
Actual detection fitting plot.

Predicted value acquisition: The method proposed in this paper was used to obtain the predictive values (represented by the y-axis in **Figure 8**).Real value acquisition: The samples were rapidly sent to the State Key Laboratory of North China Crop Improvement and Regulation for residue detection using ultra-high performance LC-tandem MS, in accordance with national standards. The pesticide residues obtained here were used as the real values (x-axis).

**Figure 9:**
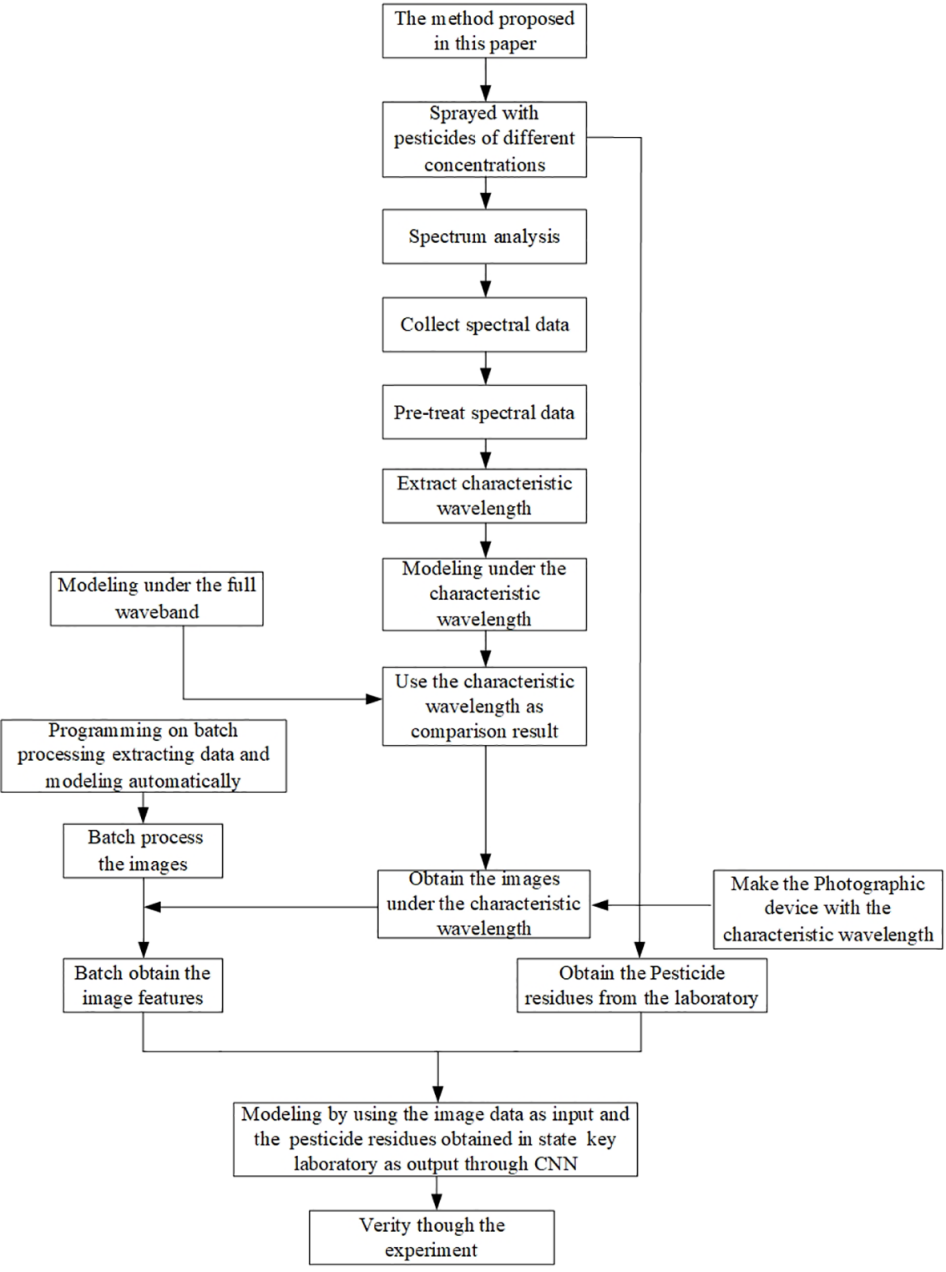
Flow chart of the method proposed in this paper.

Validation result: The comparison between the predictive values obtained by the method proposed in this paper and the real values measured by the State Key Laboratory was fitted (**Figure 8**). The detection results are shown in **Table 5**. The press was 0.489, the RMSE was 0.134mg/kg, the MAE was 0.045mg/kg, and the R^2^ value was 0.083. The accuracy was a little lower than that of the modeling process, but still met Chinese testing standards (State Bureau of Technical Supervision, 2021), proving that the proposed modeling method was highly accurate when practically applied to the detection of imidacloprid pesticides. The four steps are shown in **Figure 9** and describe the method proposed in this paper.

**Table 5:** Detection results of the validation experiment.

Parameter	Value
Press	0.489
RMSE(mg/kg)	0.134
MAE(mg/kg)	0.045
R^2^	0.883

## Discussion

The method for detecting pesticide residues on lettuce leaves proposed in this study has the advantages of both spectral detection and deep learning, and achieves rapid and accurate prediction of pesticide residues on crop leaves. As mentioned above, chemical analysis and other methods have been mostly used for traditional pesticide residue detection, but have poor real-time performance and rely on professional third-party detection institutions. In recent years, researchers at home and abroad have investigated the utility of GC, LC, spectral analysis, and other methods, for detecting pesticide residues on vegetables. However, the large size of the instruments and cumbersome data processing associated with these methods have restricted their use. Additionally, owing to the large amount of data to be extracted, the above methods are not fit for purpose in the early modeling process. In this study, the relevant published literature investigating the application and research status of technology for detecting pesticide residue in fruit and vegetables was examined. Based on investigation and analysis, this study integrated spectral analysis and machine vision methods to achieve accuracy and simplicity, and improve sample training modeling methods, paving the way for more accurate detection.

Unlike existing spectral methods for detecting pesticide residues, spectrometers were only used in the early stages of leaf characteristic information extraction and training modeling in this study. After the model was established, an improved machine vision camera was used to extract information at characteristic wavelengths, thus avoiding the need for expensive spectrometers. At the same time, detection was carried out under the characteristic wavelength. Therefore, this method is more concise than extracting spectral information in the whole band, and reduces problems such as overfitting caused by too much related data. After comparing different preprocessing methods, such as original spectral data, spectral data preprocessed by standard normalization, multivariate scattering correction, first derivative, convolution smoothing, convolution smoothing and first derivative, convolution smoothing and second derivative, and mean centralization, convolution smoothing was selected as the preprocessing method for extracting feature wavelengths in this study. Therefore, this study initially introduced the extraction process of characteristic wavelengths, and screened and selected characteristic bands (355 nm, 545 nm, 680 nm, 709 nm, 844 nm, 935 nm, and 1025 nm). Then, by comparing this method with traditional full-band modeling, the fitting coefficients of the modeling set (0.8132 and 0.8234, respectively), fitting correlation coefficients of the prediction set (0.7936 and 0.8052, respectively), and standard deviations (0.0578 and 0.0742, respectively) were obtained, verifying the above analysis. After that, by combining with coefficient correlation and PCA, it is concluded that there exists the highest correlation between reflectance value and pesticide residues and the highest score coefficient appears at 709 nm band. Therefore, 709 nm was selected as the characteristic wavelength in this study, and the corresponding characteristic light source was chosen based on the characteristic band, and thus, the image information under the characteristic light source was extracted to detect pesticide residues in leaves, making the modeling and detection process more convenient.

In this study, a detection and acquisition device was made in-house, with a filter and light source with characteristic wavelengths added to the camera, enabling the collection and detection of pesticide residue images. Compared with common spectral image detection devices, this device has stronger pertinence and is less expensive. In view of the overwhelming workload associated with the complicated image processing of traditional methods, this study has developed an automated program that integrates image preprocessing, characteristic information extraction, and training modeling. In addition, the CNN algorithm was selected as the training method for automatically extracting the characteristic information needed after the batch input of images, greatly reducing data processing. Therefore, this study reprograms the prediction program to obtain image input in the generalized prediction process and directly acquire output pesticide residues. Analysis of the detection results revealed that the determination coefficient R^2^ of the equation was 0.883, and the RMSE value was 0.134. The method proposed in this study satisfied Chinese national standards (State Bureau of Technical Supervision, 2021) in terms of accuracy, thus verifying its practical utility for detecting imidacloprid residues on crop leaves.

## Conclusion

As part of the process for detecting pesticide residues on the surface of lettuce leaves in this study, the optimal characteristic wavelength of imidacloprid pesticide residue was obtained using IR spectroscopy and found to be 709 nm. Next, the machine vision equipment was improved, and a 709-nm bandpass filter and light source were customized and installed on the camera. Photos were taken under the characteristic wavelength, and their feature information were extracted. Given the excessive modeling workload associated with the complex image processing of traditional methods, an automated program was developed to carry out image preprocessing, extraction of feature information, and construction of a training model. Moreover, the CNN algorithm was used as the training method so that the required feature information could be automatically extracted after the batch input of images, which considerably reduced data processing. Based on the our program, the prediction program was rewritten to generalize the input images in the prediction process and directly obtain output pesticide residues; as a result, pesticide residues were efficiently and rapidly detected.

The precision of the modeling test data was verified; the R^2^ value was 0.969, and the RMSE value was 0.037, verifying the high accuracy and good convergence of the proposed modeling method. When randomly collected lettuce leaf samples were tested, an R^2^ value of 0.883 and an RMSE value of 0.134 were obtained, confirming the high accuracy of the proposed modeling method when practically applied to imidacloprid detection, and satisfying Chinese national standards. Additionally, this modeling method provides accurate spectral detection, and the improved machine vision equipment, which is both accurate and pragmatic, reduces equipment costs and provides a powerful guarantee of mitigating the environmental pollution caused by excessive pesticide use in crop planting and protecting human health. However, more research on miniaturization and high efficiency is needed. Additionally, we intend to test the application of this system and method in the field with natural light, so that farmers and consumers are able to obtain relevant pesticide data.

## Data availability statement

The raw data supporting the conclusions of this article will be made available by the authors, without undue reservation.

## Author contributions

LS, XC, and XF: Conceived the idea and proposed the method. LS, XS, XF, and XC: Contributed to the preparation of equipment and acquisition of data, and wrote the code and tested the method. LS, XC, BF, and XZ: Validation results. LS, XS, and XF: Wrote the paper. LS and XC: Revised the paper. All authors read and approved the final manuscript. XC and XF are joint first authors. All authors contributed to the article and approved the submitted version.
